# Highly Efficient Thermally Activated Delayed Fluorescence Emitter Developed by Replacing Carbazole With 1,3,6,8-Tetramethyl-Carbazole

**DOI:** 10.3389/fchem.2019.00017

**Published:** 2019-01-28

**Authors:** Jia-Lin Cai, Wei Liu, Kai Wang, Jia-Xiong Chen, Yi-Zhong Shi, Ming Zhang, Cai-Jun Zheng, Si-Lu Tao, Xiao-Hong Zhang

**Affiliations:** ^1^School of Optoelectronic Science and Engineering, University of Electronic Science and Technology of China, Chengdu, China; ^2^Institute of Functional Nano and Soft Materials, Jiangsu Key Laboratory for Carbon-Based Functional Materials and Devices, Soochow University, Suzhou, China

**Keywords:** thermally activated delayed fluorescence, steric hindrance, OLED, 1, 3, 6, 8-tetramethyl-carbazole, dihedral angle

## Abstract

Carbazole (Cz) is the one of the most popular electron donors to develop thermally activated delayed fluorescence (TADF) emitters, but additional groups are generally required in the molecules to enhance the steric hindrance between Cz and electron acceptor segments. To address this issue, we replaced Cz with its derivative 1,3,6,8-tetramethyl-carbazole (tMCz) to develop TADF emitters. Two novel compounds, 6-(4-(carbazol-9-yl)phenyl)-2,4-diphenylnicotinonitrile (CzPN) and 2,4-diphenyl-6-(4- (1,3,6,8-tetramethyl-carbazol-9-yl)phenyl) nicotinonitrile (tMCzPN) were designed and synthesized accordingly. With the same and simple molecular framework, tMCzPN successfully exhibits TADF behavior, while CzPN is a non-TADF fluorophor, as the additional steric hindrance of methyl groups leads to a more twisted structure of tMCzPN. In the organic light-emitting diodes (OLEDs), tMCzPN exhibits extremely high forward-viewing maximum external quantum efficiency of 26.0%, without any light out-coupling enhancement, which is significantly higher than that of 5.3% for CzPN. These results indicate that tMCzPN is an excellent TADF emitter and proves that tMCz is a more appropriate candidate than Cz to develop TADF emitters.

## Introduction

Organic light-emitting diodes (OLEDs) have attracted great attention and are considered as next-generation solid-state lighting and displays because of their flexibility, light weight, and low-cost fabrication (Pope et al., 1963; Tang and VanSlyke, 1987; Baldo et al., 1998; Goushi et al., 2012; Uoyama et al., 2012; Zheng et al., 2013; Liu et al., 2015b,c; Li et al., 2018; Shi et al., 2018). Based on spin quantum statistics, electrical excitation generates 25% singlet excitons and 75% triplet excitons in the devices (Segal et al., 2003). OLEDs based on traditional fluorescent emitters can only utilize singlet excitons with a maximum internal quantum efficiency (IQE) of 25% (Baldo et al., 1999; Segal et al., 2003). To harvest triplet excitons, phosphorescent OLEDs, using heavy metal complexes as emitters, were developed, and successfully realized with 100% IQE, due to the strong spin-orbit coupling effect of heavy metal irons (Baldo et al., 1998; Adachi et al., 2001; Sajoto et al., 2009; Yersin et al., 2011). However, noble metal complexes also lead to expensive costs and environmental hazards, which further constrains the development of phosphorescent OLEDs (Méhes et al., 2012; Zhang et al., 2014c, 2015).

To address this issue, Adachi and co-workers firstly introduced pure organic molecules with thermally activated delayed fluorescence (TADF) characteristic as emitters for OLEDs (Uoyama et al., 2012). TADF emitters can convert non-radiative triplet excitons to radiative singlet excitons through an efficient reverse intersystem crossing (RISC) process, thus TADF-based OLEDs can also theoretically achieve 100% IQE (Endo et al., 2009). As a TADF emitter, it is key to realize an extremely small singlet and triplet energy splitting (Δ*E*_ST_) for an efficient RISC process (Peng et al., 2013). Therefore, nearly all reported TADF emitters were developed by connecting electron-donor (D) and electron-acceptor (A) segments with a highly twisted structure (Zhang et al., 2014a, 2017; Liu et al., 2016; Chen et al., 2017; Wang et al., 2017), as such a highly twisted D-A molecular framework can naturally separate the highest occupied molecular orbital (HOMO) and lowest unoccupied molecular orbital (LUMO) on the D and A segments respectively, which is the general requirement for extremely small Δ*E*_ST_s (Endo et al., 2011; Zhang et al., 2012, 2014a,b). Among the reported TADF emitters, carbazole (Cz) is one of the most popular D candidates due to its planar aromaticity, appropriate energy levels, feasible modification and good chemical stability (Uoyama et al., 2012; Liu et al., 2015a; Mounggon et al., 2015; Chan et al., 2018; Pashazadeh et al., 2018). However, with the relatively small steric hindrance of the Cz segment, Cz-A structure TADF emitters usually possess moderate dihedral angles between the Cz and A segments around 45° (Hirata et al., 2014; Zhang et al., 2018), which induces higher Δ*E*_ST_s and hinders the RISC process. Thus, to develop Cz-A structure TADF emitters, additional groups are generally required to enhance the steric hindrance between the Cz and A segments, which lead to complicated synthetic procedures and high costs.

To address this issue, Cz's derivative group 1,3,6,8-tetramethyl-carbazole (tMCz) has been proposed to replace the Cz group in the development of TADF emitters. Compared with Cz, the methyl groups at 1, 8 positions on tMCz can enhance the steric hindrance between tMCz and A segments, thus leading to more twisted structures of the compounds without other additional groups. In 2017, Adachi and co-workers developed a TADF emitter Cz-TRZ2 with tMCz group and realized a maximum EQE of 22.0% in the OLED (Cui et al., 2017). In this work, we designed and synthesized two novel compounds 6-(4-(carbazol-9-yl)phenyl)-2,4-diphenylnicotinonitrile (CzPN) and 2,4-diphenyl-6-(4-(1,3,6,8-tetramethyl-carbazol-9-yl)phenyl) nicotinonitrile (tMCzPN) by combining Cz or tMCz with electron-acceptor diphenylnicotinonitrile (Liu et al., 2015a), in a simple molecular framework. With the same molecular framework, the dihedral angles between the Cz (or tMCz) and phenylnicotinonitrile segments are 52° for CzPN and 84.9° for tMCzPN, respectively, proving that tMCz can better separate the HOMO and LUMO than Cz can. Moreover, tMCzPN successfully exhibits TADF characteristic with a small Δ*E*_ST_ of 0.10 eV, while CzPN is a non-TADF fluorophor with a large Δ*E*_ST_ of 0.32 eV. In these devices, CzPN exhibits low forward-viewing maximum current efficiency (CE), power efficiency (PE), and external quantum efficiency (EQE) of 4.7 cd A^−^ (Pope et al., 1963), 4.7 lm W^−1^, and 5.3%, respectively, consistent with common traditional fluorescent emitters. Additionally, the tMCzPN-based OLED shows high forward-viewing maximum efficiencies of 26.0% for EQE, 65.9 cd A^−1^ for CE, and 62.7 lm W^−1^ for PE, without any light out-coupling enhancement. To the best of our knowledge, these high efficiencies are among the best reported TADF-based OLEDs, proving that tMCz is a more appropriate candidate than Cz to develop TADF emitters.

## Experimental

### General Methods

^1^H nuclear magnetic resonance (NMR) and ^13^C NMR spectral data were obtained by using an AVANCZ spectrometer. Mass spectral (MS) data were measured using a Finnigan 4021C gas chromatography mass spectrometry instrument. Absorption and Photoluminescence (PL) spectra were obtained using a Hitachi UV-vis spectrophotometer U-3010 and a Hitachi fluorescence spectrometer F-4600, respectively. Cyclic voltammetry (CV) measurements were performed using a CHI660E electrochemical analyzer, while the oxidation potential of saturated calomel electrode (SCE) relative to the vacuum level is calibrated to be 4.56 eV in dimethylformamide (DMF). Transient PL spectra were obtained using Edinburgh Instruments FLS980 spectrometer. The photoluminescence quantum yields (PLQYs) of mixed DPEPO (bis[2-(di-(phenyl)phosphino)-phenyl]ether oxide) solid films were investigated using a QY-2000 fluorescence spectrometer and estimated via a F-3018 Integrating Sphere. Thermogravimetric analysis (TGA) and differential scanning calorimetry (DSC) measurements were performed using a TAQ 500 thermogravimeter and a NETZSCH DSC204 instrument in N_2_, respectively.

### Synthesis

The commercially available reagents and materials were used directly without purification.

### (E)-1-(4-bromophenyl)-3-phenylprop-2-en-1-one

A mixture of 1-(4-bromophenyl)ethan-1-one (2.0 g, 10 mmol) and benzaldehyde (2.12 g, 20 mmol) was stirred in 20 ml of ethanol and a 10% NaOH solution for 30 min at room temperature. The reaction mixture was allowed to stand for 1 h and then filtered and recrystallized from ethanol to obtain a light yellow powder (E)-1-(4-bromophenyl)-3-phenylprop-2-en-1-one (2.5 g, 87% yield). The crude product was dried in a vacuum oven directly for the next step.

### 6-(4-bromophenyl)-2,4-diphenylnicotinonitrile

3-oxo-3-phenylpropanenitrile (1.45 g, 10 mmol), (E)-1-(4-bromophenyl)-3-phenylprop-2-en-1-one (2.87 g, 10 mmol) and anhydrous ammonium acetate (4.01 g, 52 mmol) was dissolved in glacial acid (8 mL). The mixture was refluxed under stirring for 2 h at 110°C. After cooling to room temperature, the reaction was neutralized using a 10% NaOH solution and extracted with dichloromethane. The dichloromethane phase was then dried with anhydrous sodium sulfate. After evaporation of the solvent, the crude product was purified through silica gel column chromatography, using 1:3 dichloromethane/petroleum as eluent to obtain a white solid powder 6-(4-bromophenyl)-2,4-diphenylnicotinonitrile (1.0 g, 24.3% yield). ^1^H NMR (600 MHz, Chloroform-*d*) δ 8.09–8.00 (m, 4H), 7.79 (s, 1H), 7.73 – 7.61 (m, 4H), 7.56 (dt, *J* = 7.7, 5.8 Hz, 6H). ^13^C NMR (151 MHz, Chloroform-*d*) δ 162.62, 158.05, 155.80, 137.95, 136.74, 136.51, 132.32, 130.32, 130.16, 129.48, 129.18, 128.79, 128.69, 125.43, 118.50, 117.70, 104.81. MS (EI) m/z: [M]+ calcd for C_24_H_15_BrN_2_, 411.30; found, 411.05.

### 6-(4-(Carbazol-9-yl)phenyl)-2,4-diphenylnicotinonitrile (CzPN)

A mixture of 6-(4-bromophenyl)-2,4-diphenylnicotinonitrile (0.411 g, 1 mmol), Cz (0.200 g, 1.2 mmol), sodium tert-butoxide (0.192 g, 2 mmol), tris(dibenzylideneacetone)dipalladium (0.018 g, 0.02 mmol) and tri-tert-butylphosphine tetrafluoroborate (0.023 g, 0.008 mmol) was added into toluene (10 mL) under N_2_ and then refluxed at 110°C for 12 h. After cooling to room temperature, the mixture was filtered with diatomite. The solvent of the filtrate was removed under reduced pressure. The crude product was purified through silica gel column chromatography, using 1:3 dichloromethane/petroleum as eluent to obtain a green solid powder (0.425g, 86% yield). ^1^H NMR (600 MHz, Chloroform-*d*) δ 8.45 – 8.42 (m, 2H), 8.16 (d, *J* = 7.7 Hz, 2H), 8.11 – 8.07 (m, 2H), 7.92 (d, *J* = 1.6 Hz, 1H), 7.75 (ddd, *J* = 11.0, 7.5, 1.7 Hz, 4H), 7.63 – 7.54 (m, 6H), 7.51 (d, *J* = 8.2 Hz, 2H), 7.46 – 7.42 (m, 2H), 7.32 (t, *J* = 7.4 Hz, 2H). ^13^C NMR (151 MHz, Chloroform-*d*) δ 162.56, 158.15, 155.66, 140.44, 139.83, 137.88, 136.66, 136.27, 130.17, 130.02, 129.36, 129.11, 129.04, 128.67, 128.56, 127.17, 126.09, 123.64, 120.41, 120.32, 118.61, 117.62, 109.76, 104.55. MS (EI) m/z: [M]+ calcd for C_36_H_23_N_3_, 497.60; found, 497.19.

### 2,4-diphenyl-6-(4-(1,3,6,8-tetramethyl-carbazol-9-yl)phenyl)nicotinonitrile (tMCzPN)

tMCzPN was synthesized according to the same procedure as CZN,by using tMCz instead of Cz. After cooling to room temperature, the mixture was filtered with diatomite. The solvent of filtrate was removed under reduced pressure; the crude product was purified through silica gel column chromatography using 1:3 dichloromethane/petroleum as eluent to obtain a yellow solid powder (0.25 g, 45% yield). ^1^H NMR (600 MHz, Chloroform-*d*) δ 8.32 (d, *J* = 8.4 Hz, 2H), 8.08 (dd, *J* = 8.0, 1.6 Hz, 2H), 7.95 (s, 1H), 7.80–7.69 (m, 4H), 7.66 – 7.53 (m, 8H), 6.92 (s, 2H), 2.48 (s, 6H), 1.93 (s, 6H). ^13^C NMR (151 MHz, Chloroform-*d*) δ 162.75, 158.04, 155.92, 144.86, 139.51, 138.07, 137.75, 136.85, 132.03, 130.46, 130.44, 130.25, 129.58, 129.36, 129.26, 128.87, 128.82, 127.52, 124.45, 121.36, 118.98, 118.05, 117.78, 104.95, 21.32, 19.82. MS (EI) m/z: [M]+ calcd for C_40_H_31_N_3_, 553.71; found, 553.25.

### Device Fabrication and Measurements

ITO-coated glass substrates with a sheet resistance of 30 Ω per square, were cleaned with acetone and ethanol, then washed with deionized water for 5 min, and then oven-dried at 120°C and treated with UV-ozone for 5 min. The cleaned substrates were then moved into the vacuum evaporation chamber. The organic layers were deposited onto the substrates through thermal evaporation under 1 × 10^−6^ Torr with the rate of 1–2 Å s^−1^. The rate was 0.1 Å s^−1^ for LiF, and 10 Å s^−1^ for Al. The electroluminescence (EL) spectra and CIE color coordinates were measured using a Spectrascan PR650 instrument. The current density-voltage characteristics were obtained using a Keithley 2400 Source Meter controlled by a computer. The EQE was calculated from the current density, luminance, and EL spectrum, assuming a Lambertian distribution (Okamoto et al., 2001).

## Results and Discussion

### Synthesis

**Scheme 1:**
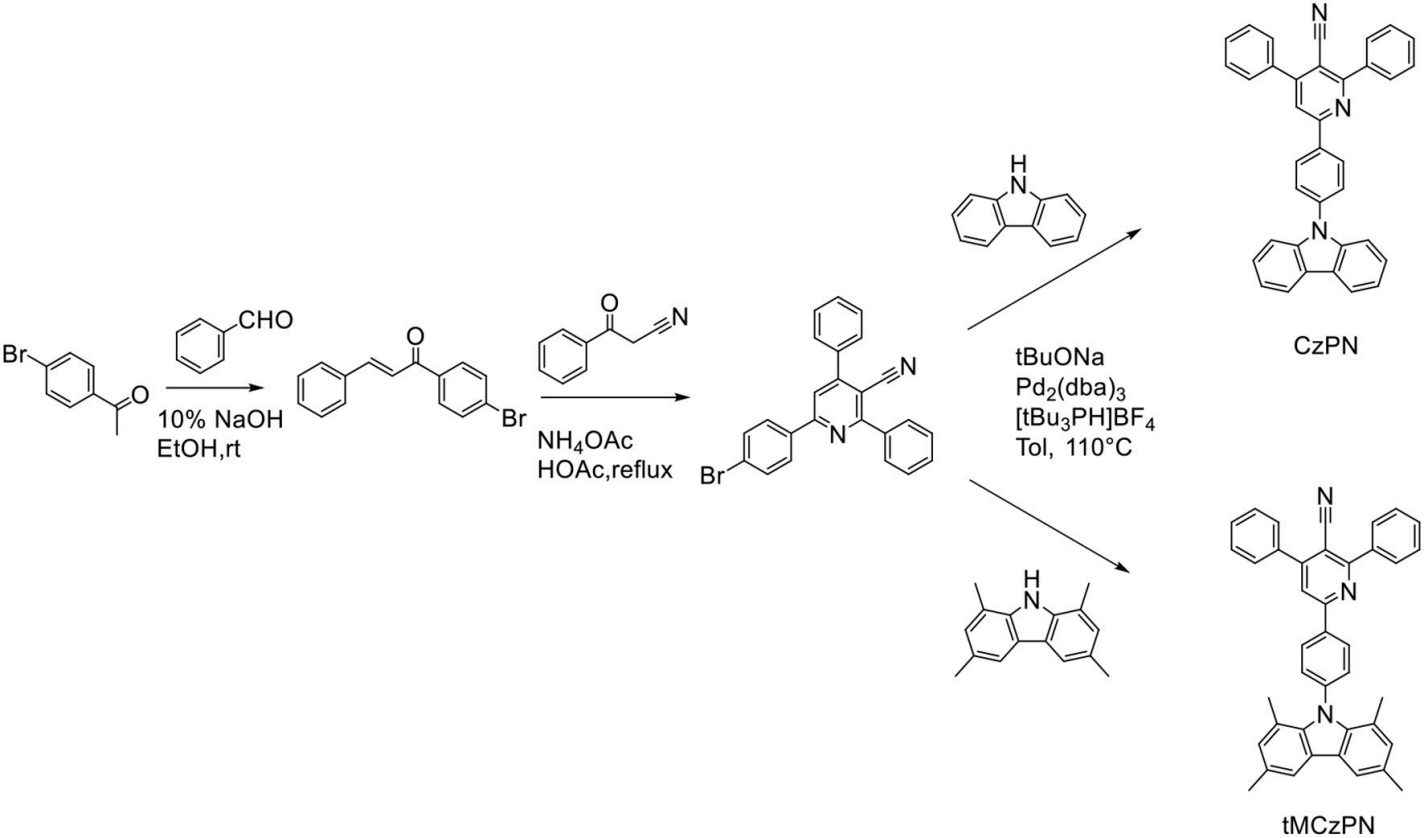
Synthetic routes and molecular structures of CzPN and tMCzPN.

As shown in Scheme 1, both two compounds were synthesized through three steps. The (E)-1-(4-bromophenyl)-3-phenylprop-2-en-1-one was synthesized by aldol reaction between benzaldehyde and 1-(4-bromophenyl)ethan-1-one. And the cyanopyridine derivative was synthesized by cyclizing a pyridine ring between 3-oxo-3-phenylpropanenitrile and (E)-1-(4-bromophenyl)-3-phenylprop-2-en-1-one with ammonium acetate as the source of nitrogen. Then, the final products were synthesized through the Buchwald-Hartwig coupling reaction between cyanopyridine derivative and the corresponding Cz derivatives. The chemical structure of CzPN and tMCzPN were characterized and confirmed by ^1^H NMR and ^13^C NMR spectroscopies and mass spectrometry. Moreover, CzPN and tMCzPN were purified by sublimation before further characterizations.

### Theoretical Calculations and Electrochemical Properties

**Figure 1:**
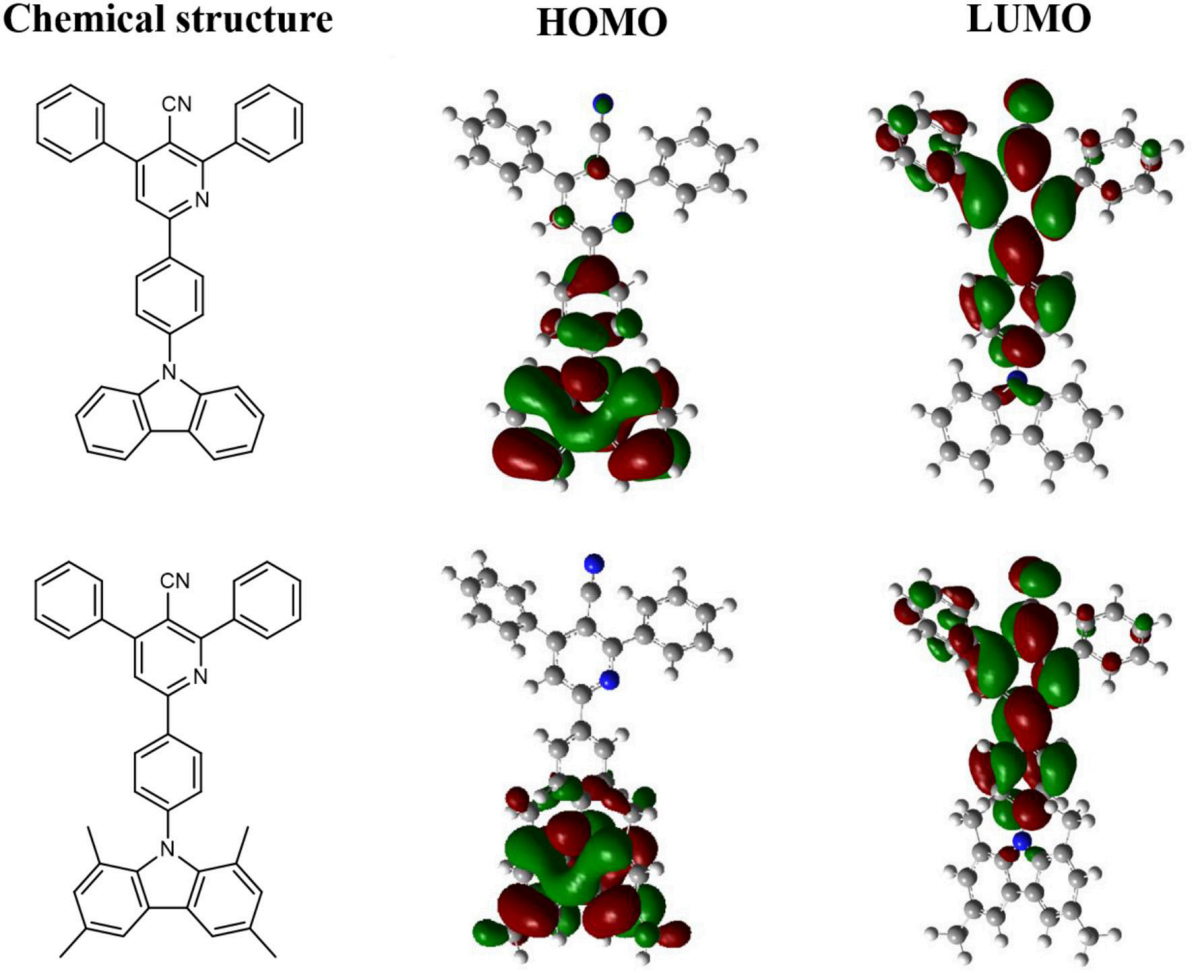
Chemical structures, HOMO and LUMO distributions of CzPN and tMCzPN.

To analyze the relationships between the structures and properties of CzPN and tMCzPN at the molecular level, we performed the density function theory (DFT) calculation for both compounds at the B3LYP/6-31G(d) level. The optimized ground-state conformations and HOMO and LUMO distributions of the two compounds are shown in Figure 1. In the CzPN molecule, the dihedral angle between the Cz and phenylnicotinonitrile segments was optimized to 52°, which is close to other reported common Cz-A structure compounds, without steric hindrance between the D and A segments (Choi et al., 2017; Rajamalli et al., 2017; Liang et al., 2018) . Caused by such insufficient twist, both the HOMO and LUMO of CzPN are extended to the central benzene bridge. The obvious overlap between HOMO and LUMO leads to an evident conjugation and a large Δ*E*_ST_ for CzPN, which suppresses the TADF behavior. Reversely, due to the large hindrance of the two methyl groups at 1, 8 positions on the Cz, tMCzPN possesses a nearly vertical dihedral angle between the Cz and phenylnicotinonitrile segments of 84.9°. The HOMO is mainly confined on the tMCz segment and the LUMO is located on the phenylnicotinonitrile unit, realizing a nearly full separation between HOMO and LUMO. Thus, by replacing Cz with tMCz, tMCzPN successfully possesses a more twisted molecular structure and is predicted to have an extremely small Δ*E*_ST_ and the TADF characteristic theoretically.

**Figure 2:**
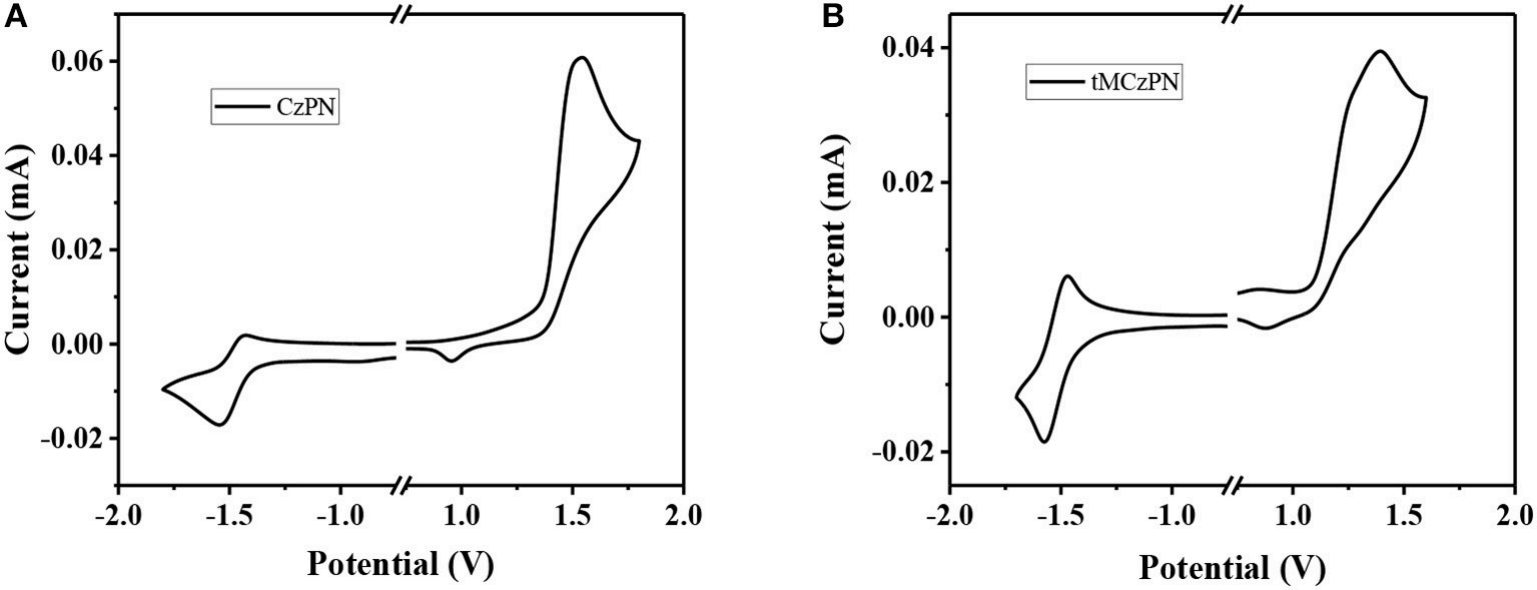
Cyclic voltammograms of **(A)** CzPN and **(B)** tMCzPN in DMF recorded with a scan rate of 0.05 V/s. The HOMO and LUMO levels are calculated with the equation: HOMO = – *e*(*E*_*ox*_ + 4.56), LUMO = – *e*(*E*_*red*_ + 4.56). *E*_*ox*_, *E*_*red*_ are the onsets of oxidation and reduction curves.

We further investigated the electrochemical properties of both compounds by CV in DMF. As shown in Figure 2, from the onsets of oxidation curves, the HOMO energy levels of CzPN and tMCzPN are estimated to be −5.93 and −5.66 eV, respectively. As the electron-donating ability of the four methyl groups, tMCz has a much higher HOMO energy level than Cz. Whereas, from the onsets of reduction curves, the LUMO energy levels of CzPN and tMCzPN are calculated to be of similar values of −3.16 and −3.13 eV, respectively.

### Thermal Properties

**Figure 3:**
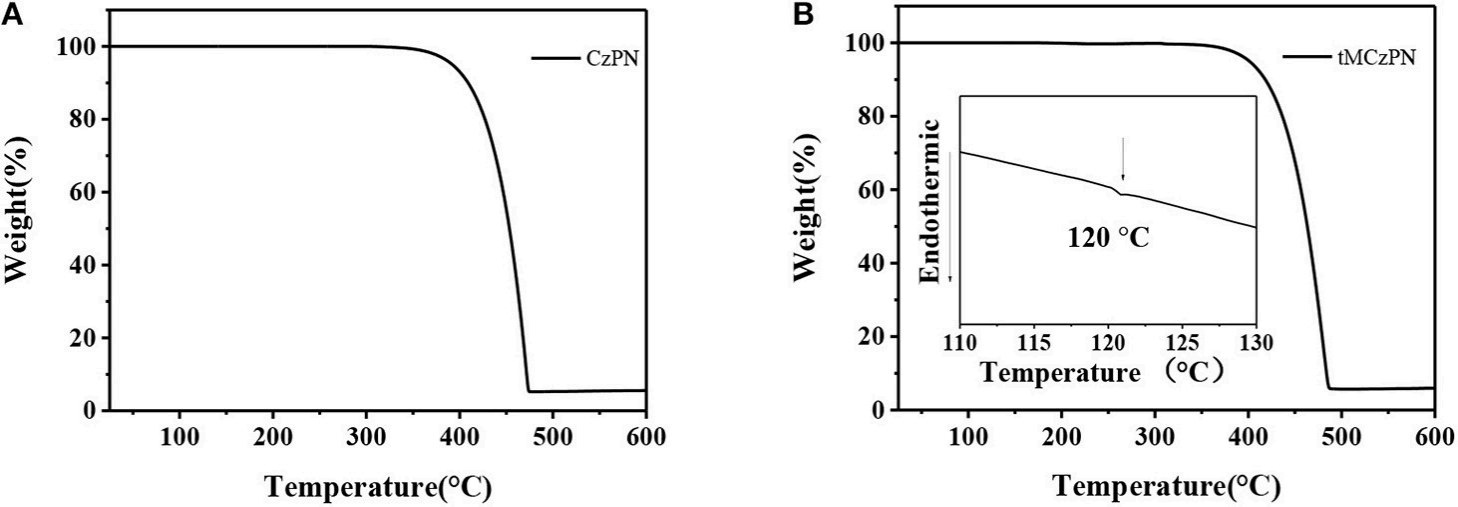
TGA and DSC measurements of **(A)** CzPN and **(B)** tMCzPN recorded with a heating rate of 10°C/min.

The thermal properties of CzPN and tMCzPN were characterized by TGA and DSC measurements under a nitrogen atmosphere. As shown in Figure 3, two compounds exhibit high decomposition temperatures (T_d_s) (corresponding to 5.0 % weight loss) of 392°C for CzPN and 400°C for tMCzPN, respectively, suggesting that both are capable of vacuum purification and evaporation. The glass transition temperature (T_g_) of tMCzPN is determined to be 120°C, and no glass transition was observed for CzPN from 25 to 250°C. The high T_g_ of tMCzPN would be beneficial to its morphological stability and reduce the phase separation rate of the guest-host system.

### Photophysical Properties

**Figure 4:**
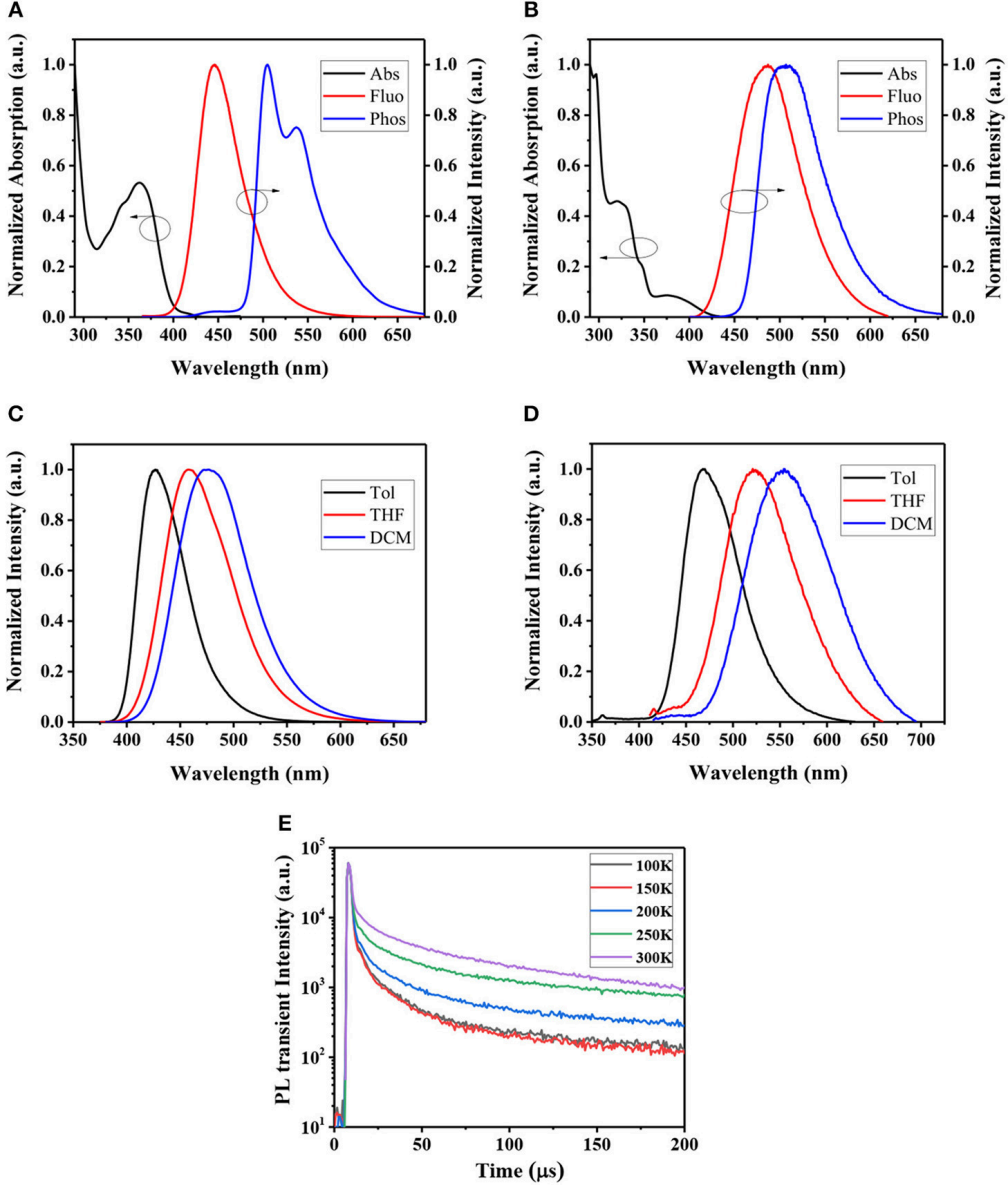
UV-Vis absorption spectra in toluene, fluorescence spectrum in DPEPO film at room temperature, and phosphorescence spectra in DPEPO film at 77K of **(A)** CzPN and **(B)** tMCzPN. Fluorescence spectra of **(C)** CzPN and **(D)** tMCzPN in different solvent (Tol, toluene; DCM, dichloromethane; and THF, tetrahydrofuran). **(E)** Temperature-dependent transient PL decay spectra of tMCzPN doped in DPEPO film by exciting at 300 nm (20 wt.%).

Room temperature UV-Vis absorption spectra of CzPN and tMCzPN in toluene are shown in Figure 4. At the long-wavelength region, both compounds show broad absorption bands with peaks at 362 and 375 nm, respectively for CzPN, and tMCzwhich are assigned to the intramolecular charge transfer (ICT) transition from the electron donor Cz to the electron acceptor phenylnicotinonitrile. Additionally, the ICT absorption of tMCzPN was much weaker than that of CzPN, which is ascribed to its better separation between the HOMO and LUMO as shown in Figure 2. With the ICT transition characteristic, both compounds exhibit obvious solvatochromic effects in variated solvents as shown in Figures 4C,D. From low-polar toluene to high-polar dichloromethane, the emission peak of CzPN is red-shifted from 427 to 477 nm, while the emission peak of tMCzPN is red-shifted from 468 to 554 nm. As the solvatochromic effect is related to structure relaxation of the ICT molecule, CzPN, which has a higher molecular restriction due to stronger conjugation between the D and A segments, exhibits a much weaker red-shift compared to tMCzPN accordingly.

The PL spectra of the two compounds doped in DPEPO films were further studied. At room temperature, CzPN and tMCzPN both exhibit shapeless fluorescent spectra peaked at 446 and 487 nm, respectively. Due to the stronger electron-donating ability of tMCz, the fluorescence of tMCzPN is evidently red-shifted. At 77 K, we obtained the phosphorescent spectra of two compounds. CzPN showed a local-excited feature phosphorescence with two sharp peaks, because the extended conjugation lowers the triplet energy level of Cz. While tMCzPN exhibits a shapeless and board phosphorescence, suggesting its T_1_ state is still ICT characteristic. From the peaks of fluorescent and phosphorescent spectra, the S_1_ and T_1_ energy levels are estimated to be 2.78 and 2.46 eV for CzPN, and 2.55 and 2.45 eV for tMCzPN. As listed in Table 1, the Δ*E*_ST_s of CzPN and tMCzPN are estimated to be 0.32 and 0.10 eV, respectively. With a large Δ*E*_ST_ and different features of S_1_ and T_1_ states, the up-conversion from triplet to singlet excitons will be inhibited, making it difficult for CzPN to possess TADF characteristic. Reversely, the small Δ*E*_ST_ of 0.10 eV will lead tMCzPN to realize an efficient RISC process and exhibit TADF behavior. In DPEPO films at room temperature, the PLQYs of CzPN and tMCzPN were measured to be 93.3 and 70.5%, respectively. With a significant overlap between HOMO and LUMO, the fluorescence process of singlet excitons is efficient for CzPN, and it can be an excellent conventional fluorescent emitter. Additionally, tMCzPN also realizes quite a high PQLY for TADF emitters.

**Table 1:** Key physical properties of two compounds.

**Compounds**	**λ_abs_ (nm) ^a^**	**λ_fluo_ (nm) ^b^**	**λ_phos_ (nm) ^c^**	**ΔE_ST_ (eV) ^d^**	**HOMO (eV) ^e^**	**LUMO (eV) ^f^**	**T_d_/T_g_ (^°^C)**	**PLQY (%)**
CzPN	362	446	505	0.32	−5.93	−3.16	392/	93.3
tMCzPN	375	487	507	0.10	−5.66	−3.13	400/120	70.5

a *Measured in toluene solution at room temperature*.

b *Measured in DPEPO film at room temperature*.

c *Measured in DPEPO film at 77 K*.

d *Estimated from the peak of fluorescence and phosphorescence spectra*.

e *Calculated from the onset of oxidation potential*.

f *Calculated from the onset of reduction potential*.

To further prove their TADF characteristics, the transient PL decays of the two compounds doped into DPEPO films were measured. In the order of a microsecond, no delayed component was observed for CzPN, indicating its non-TADF characteristic. While for tMCzPN, a delayed decay with a lifetime of 14.29 μs was obtained at room temperature. We also measured the temperature-dependent transient PL decays of the tMCzPN doped DPEPO film from 100 to 300 K. As shown in Figure 4E, the delayed component clearly enhanced with the increasing temperature, due to the acceleration of the up-conversion from triplet to singlet excitons by thermal activation, which directly demonstrated the TADF characteristic of tMCzPN. By replacing the common Cz with tMCz, CzPN, and tMCzPN exhibit significant differences in some key photophysical properties. Thus, compared to Cz, tMCz is more convenient to develop TADF emitters.

### Electroluminescence Properties

To investigate the EL performance of two compounds, devices with structures of ITO/TAPC (35 nm)/TCTA (10 nm)/CzSi (10 nm)/DPEPO: CzPN or tMCzPN (20 wt%) (20 nm)/TmPyPb (40 nm)/LiF (1 nm)/Al were fabricated. Herein, indium tin oxide (ITO) was the anode, 4,4′-cyclohexylidenebis[N,N-bis(4-methylphenyl)aniline (TAPC) and 4,4′,4″-tris(carbazolyl)triphenylamine (TCTA) were the hole-transporting layers, 9-(triphenyl-silyl)-9H-carbazole (CzSi) was the exciton-blocking layer, 1,3,5-tri(m-pyrid-3-yl-phenyl)benzene (TmPyPb) was electron-transporting, hole-blocking, and exciton-blocking layer, LiF was the electron injection layer, and Al was the cathode, respectively. CzPN or tMCzPN doped DPEPO was used as the emitting layers, and the doping concentration was optimized to 20 wt%.

**Figure 5:**
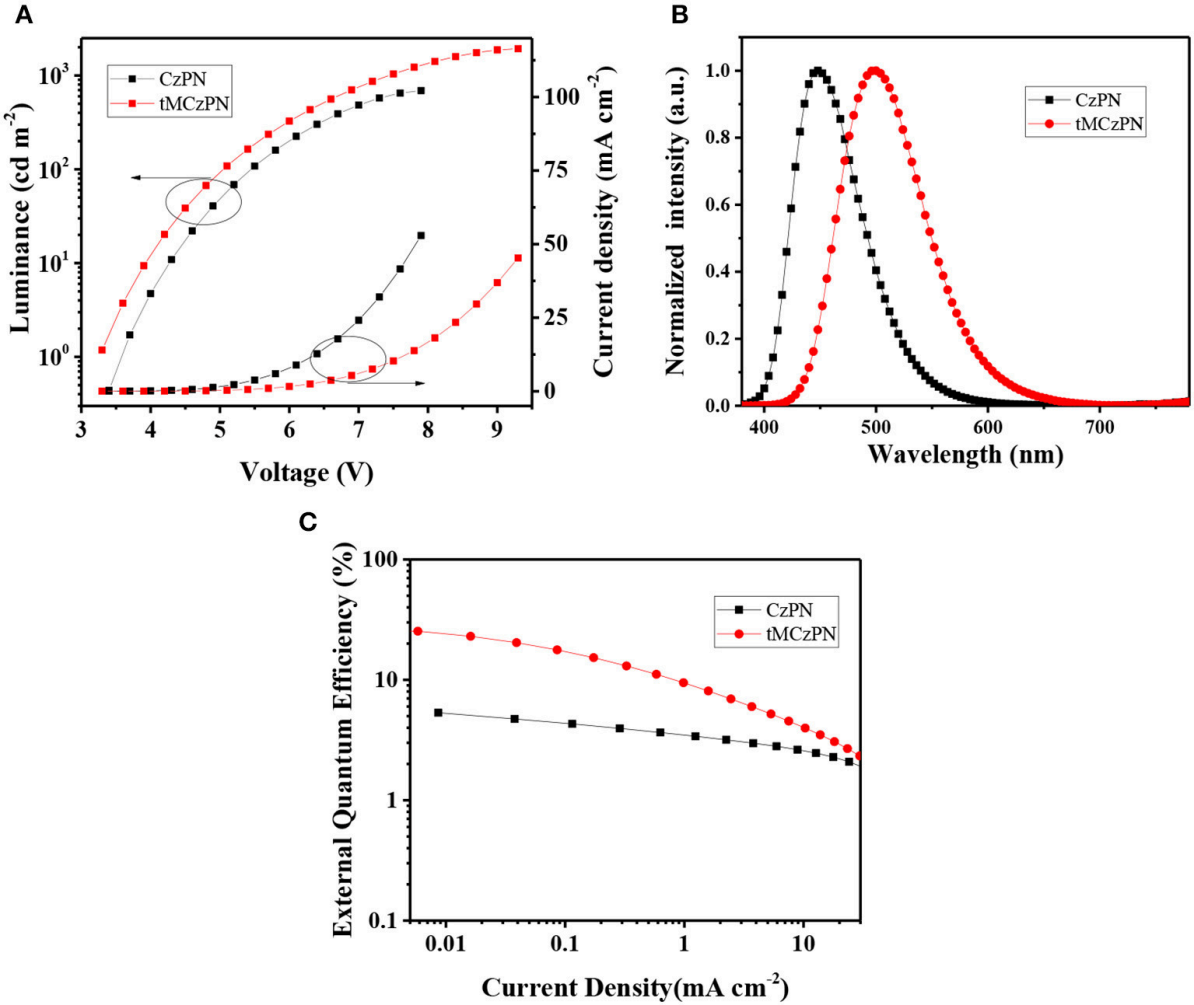
**(A)** Current density-voltage-luminance characteristics, **(B)** EL spectra, and **(C)** Current density-EQE characteristics of the devices based on CzPN and tMCzPN.

As shown in Figure 5 and listed in Table 2, both devices show close turn on voltages of 3.4 V and 3.3 V, respectively for CzPN and tMCzPN. The similar turn on voltages should be attributed to the use of the same host material, which is consistent with the results of the reported devices using DPEPO as the host. In the devices, CzPN exhibits a stable blue emission with a peak at 448 nm and a CIE coordinate of (0.15, 0.11), while tMCzPN shows a stable cyan emission with a peak at 500 nm and a CIE coordinate of (0.20, 0.42). The red-shift between two EL spectra should be mainly ascribed to the stronger electron-donating ability of tMCz compared with Cz, and is consistent with their PL spectra in DPEPO films. Additionally, from the EL spectra, the full-width at half-maximum is 72 nm for CzPN, evidently narrower than 90 nm for tMCzPN. This is because CzPN has a higher conjugation between the D and A segments, and thereby possesses a stronger molecular restriction to confine structural relaxation. The maximum forward-viewing efficiencies of CzPN-based OLED are 4.7 cd A^−1^ for CE, 4.7 lm W^−1^ for PE, and 5.3% for EQE, respectively. The EQE of 5.3% is consistent with the theoretical limitation of the OLEDs based on traditional fluorescent emitters, demonstrating the non-TADF characteristic of CzPN yet again. Without any light out-coupling enhancement, the tMCzPN-based device exhibits extremely high forward-viewing maximum CE, PE and EQE of 65.9 cd A^−1^, 62.7 lm W^−1^ and 26.0%, respectively. To the best of our knowledge, such a high EQE of 26.0% is among the best performance for TADF-based OLEDs. Thus, by simply replacing common Cz with tMCz, the non-TADF fluorophor CzPN is successfully transformed to an excellent TADF emitter tMCzPN, indicating that tMCz is a more appropriate candidate than Cz, for developing TADF emitters.

**Table 2:** Electroluminescence properties of the devices.

**Emitters**	**V_on_ (V)^a^**	**CE_max_ (cd A^−1^) ^b^**	**PE_max_ (lm W^−1^) ^c^**	**EQE_max_ (%)^d^**	**Peak (nm)**	**FWHM (nm)^e^**	**CIE**
CzPN	3.4	5.1	4.7	5.3	448	72	(0.15, 0.11)
tMCzPN	3.3	65.9	62.7	26.0	500	90	(0.20, 0.42)

a *Turn-on voltage, measured at the luminance of 1 cd m^−1^*.

b *Maximum current efficiency*.

c *Maximum power efficiency*.

d *Maximum external quantum efficiency*.

e *Full-width at half-maximum*.

## Conclusion

Due to the insufficient steric hindrance of Cz, additional groups are generally required to enhance the separation between HOMO and LUMO for Cz-based TADF emitters, resulting in complicated synthetic procedures and high costs. To address this issue, we replaced Cz with its derivative tMCz to develop TADF emitters, and designed and synthesized two novel compounds CzPN and tMCzPN accordingly. With the same and simple molecular framework, two compounds exhibit evident differences due to the additional methyl groups on tMCz. tMCzPN possesses a more twisted molecular structure and successfully realizes the TADF characteristic with a small Δ*E*_ST_ of 0.10 eV, while CzPN is a non-TADF fluorophor. In the devices, tMCzPN exhibits an extremely high forward-viewing maximum EQE of 26.0%, without any light out-coupling enhancement, which is significantly higher than that of 5.3% for CzPN. These results indicate that tMCzPN is an excellent TADF emitter and proves that tMCz is a more appropriate candidate than Cz for developing TADF emitters with a simple molecular framework.

## Author Contributions

J-LC and WL contributed equally to this work. C-JZ, S-LT, and X-HZ designed whole work. J-LC, WL, and J-XC synthesized the organic compounds. J-LC, KW, and Y-ZS characterize the physical properties of compounds. WL and MZ fabricated and optimized the devices. J-LC and WL wrote the paper with support from C-JZ, S-LT, and X-HZ. All authors contributed to the general discussion.

### Conflict of Interest Statement

The authors declare that the research was conducted in the absence of any commercial or financial relationships that could be construed as a potential conflict of interest.
